# The impact of physical activities on cognitive function among older adult populations: evidence from China

**DOI:** 10.3389/fpubh.2025.1562747

**Published:** 2025-04-11

**Authors:** Xiao Chen, Qishun Yang, ZhiYun Liu

**Affiliations:** ^1^School of Sports Training, Tianjin University of Sport, Tianjin, China; ^2^Department of Physical Education, Zhongnan University of Economics and Law, Wuhan, China; ^3^School of Physical Education and Equestrian, Wuhan Business University, Wuhan, China

**Keywords:** physical activity, middle-aged and older adults, cognition, CHARLS, fixed effects regression

## Abstract

**Objective:**

This study investigates the impact of physical activity on cognitive function in Chinese middle-aged and older adult individuals, examining the relationship between different intensities of physical activity and cognitive function, as well as the effect of physical activity on various types of cognitive function.

**Method:**

A total of 27,529 samples were selected from the China Health and Retirement Longitudinal Study (CHARLS) data from 2011 to 2018 for regression analysis. Multidimensional fixed effects estimation was used to validate the impact of physical activity on cognitive function in middle-aged and older adults, analyzing differences in the intensity of physical activity and types of cognitive function. The empirical results were further tested using methods such as propensity score matching (PSM). Additionally, this paper conducted heterogeneity analyses by gender, place of residence, presence of children, and marital status.

**Results:**

Physical activity had a positive impact on cognitive function among middle-aged and older adults in China by improving their cardiovascular health, and this result held true regardless of gender, rural or urban residence, presence of children, or marital status. This conclusion was supported by both alternative explanatory variables and results from propensity score matching. Further research found that physical activity improves immediate memory and delayed recall among middle-aged and older adults. Moderate physical activity enhances the health of the older adult population, whereas excessive physical activity may impair cognitive function in middle-aged and older adults.

**Conclusion:**

Our study found that PA can effectively promote the improvement of cognitive function in middle-aged and older adults, with this conclusion still holding true in older populations. Such physical activity should be moderate, because vigorous physical activity may impair cognitive function in middle-aged and older people. The study conclusions play a significant role in promoting physical activity, delaying cognitive decline, achieving active aging, and reducing the burden of informal caregiving.

## 1 Introduction

With the intensification of global aging, how to improve the well-being of the older adult and achieve healthy aging has become a concern for researchers. According to the World Health Organization (WHO), by 2050, the world’s population of 60 and over is expected to reach two billion, up from 900 million in 2015 (1). Recent data from the seventh national population census reveals a striking statistic: in 2020, the cohort aged 60 and above reached 264 million, representing 18.7% of China’s total population. The growth trend of the older adult population has brought serious challenges to China’s economic development and social security (2). Among them, the decline of cognitive function in the older adult seems to be an inevitable consequence of aging (3).

Mild cognitive impairment (MCI) among older adults has emerged as a critical public health challenge in aging societies (4). As a clinical transitional phase between normal cognition and dementia, MCI not only signifies substantial deterioration in individual cognitive functioning but is also recognized as a pivotal window for delaying dementia progression (5). Current evidence indicates that cognitive decline involves not only core domain impairments but is frequently accompanied by neuropsychiatric symptom clusters such as anxiety and depression, along with significant deterioration in instrumental activities of daily living (IADL) (6).

Cognitive impairments are prevalent among the older adult, including memory decline, attention deficits, deterioration of executive functions, and reduced spatial visual abilities (7). The decline in cognitive function significantly affects the self-care abilities of the older adult, impacting their quality of life and increasing the burden of care for families and society (8). Severe cognitive decline requires long-term physical care, reducing the quality of life in later years and profoundly affecting the healthy life expectancy of the older adult population (9). Therefore, exploring the causes of cognitive impairment is of great significance for enhancing the cognitive health of the older adult and improving their overall well-being (10).

The relationship between physical activity (PA) and cognitive function in older adults has been a subject of extensive research over the past few decades (11). As the global population ages, the prevalence of cognitive decline and dementia is increasing, posing significant challenges to public health and quality of life (12). Physical activity has been proposed as a potentially modifiable risk factor that could help maintain and enhance cognitive function in older adults. However, the evidence regarding the specific nature and extent of this relationship remains inconsistent and complex (13). Several systematic reviews and meta-analyses have been conducted to synthesize the existing literature on PA and cognitive function in older adults. A Cochrane review by Angevaren et al. (14) concluded that physical activity can improve cognitive function in older people without known cognitive impairment. Similarly, a meta-analysis by Colcombe et al. (15) found that fitness training has a positive effect on the cognitive function of older adults. More recently, a systematic review and meta-analysis by Iso-Markku et al. (11) examined the longitudinal associations of physical activity with cognition and found that physical activity was associated with better late-life cognition, although the association was weak. The type and intensity of physical activity have also been identified as important factors in determining its impact on cognitive function (16). For instance, a study by Erickson et al. (17) demonstrated that exercise training increases the size of the hippocampus and improves memory in older adults. Furthermore, a review by Erickson et al. (18) highlighted the importance of meeting 24-h movement guidelines for cognitive health. However, the specific cognitive domains that benefit from physical activity may vary. For example, a study by Hötting & Röder (19) found that physical activity was associated with global cognition and specific cognitive domains such as episodic memory and verbal fluency. The role of sedentary behavior in cognitive function has also been explored. A systematic review by Barha et al. (20) suggested that there is a significant association between sedentary behavior and cognitive function. Moreover, a study by Covenant et al. (21) predict cognitive performance from actigraphy data and found preliminary evidence of the interactive association between physical activity, sedentary behavior, and sleep with cognitive health. The use of objective measures such as accelerometers has provided more reliable data on the relationship between physical activity and cognitive function. A cross-sectional study by Feng et al. (22) found that accelerometer-assessed physical activity was positively associated with better processing speed in the youngest-old adults. However, the directionality of these associations remains to be determined. In conclusion, while there is a growing body of evidence suggesting that physical activity is associated with better cognitive function in older adults, the specific mechanisms and conditions under which these benefits occur remain to be fully understood. Further research is needed to clarify the role of different types and intensities of physical activity.

The purpose of this study is to: (i) explore the relationship between physical activity and cognitive function in middle-aged and older adults; (ii) estimate the trends in cognitive function changes among middle-aged and older adults at different levels of physical activity; and (iii) propose policy recommendations to improve cognitive function in middle-aged and older adults and achieve healthy aging.

## 2 Method

### 2.1 Data sources

The samples of this study were derived from the 2011, 2013, 2015 and 2018 China Health and Retirement Longitudinal Study (CHARLS) data sets. Considering the impact of the pandemic on individual behavior and health, we did not include CHARLS 2020 data in this study. CHARLS data is a follow-up survey for middle-aged and older adult groups over the age of 45 in China, covering 28 provinces and 450 village-level units in China. CHARLS sampling is very advanced, with good randomness and representativeness, which makes its data a good representative of the situation of middle-aged and older adult groups in China (23). Only samples aged 45 and above were selected in the study, and samples with key variables were excluded. Thus, 27,529 valid observations were finally obtained.

### 2.2 Assessment of physical activity

This paper measures participation in different intensity types of physical activity (PA) based on responses in the CHARLS questionnaire, setting participation in at least one type of physical activity as 1 and no participation in any type of physical activity as 0, to explore the impact of physical activity on individual cognitive function. Additionally, this paper similarly handles different intensity types of physical activity for subsequent in-depth research.

### 2.3 Assessment of cognition

Past research has indicated that memory capacity is an important content for assessing an individual’s cognitive status (24). This paper constructs a cognitive function variable based on the immediate and delayed recall of 10 words from the CHARLS questionnaire. Each time the sample recalls a word, they score 1 point, with a minimum of 0 and a maximum of 20. Additionally, this paper uses the self-assessment of memory from the sample to construct an alternative explanatory variable, setting samples who self-assess their memory as 0 to represent poor memory, otherwise as 1 to represent good memory. We also use respondent’s number of correct subtractions in the serial 7’s test in order to comprehensively value their cognition function.

### 2.4 Covariates

Our article incorporates participants’ demographic characteristics as control variables to enhance the model’s explanatory power, with assessed variables including: age (continuous, at least 45 years old), gender (male, female). Education level (primary education and below, high school and vocational education, higher education), marital status (married, other), place of residence (rural, urban), smoking status, drinking status, self-assessed health status (good, fair, poor), number of living children, and presence of children. Additionally, this article fixes regional and temporal effects based on the sample province and survey year, and clusters at the village level to enhance the model’s explanatory power (25).

Descriptive statistics of variables are shown in Table 1.

**Table 1:** Descriptive statistics.

Var name	Obs	Mean	SD	Min	Max
cognition	27,529	7.693	3.741	0.000	20.000
PA	27,529	0.915	0.278	0.000	1.000
age	27,529	59.932	9.035	45.000	108.000
gender	27,529	0.508	0.500	0.000	1.000
health	27,529	3.070	0.967	1.000	5.000
marriage	27,529	0.893	0.309	0.000	1.000
education	27,529	1.171	0.432	1.000	3.000
rural	27,529	0.745	0.436	0.000	1.000
smoking	27,529	0.443	0.497	0.000	1.000
drinking	27,529	0.474	0.499	0.000	1.000
hchild	27,529	2.569	1.295	1.000	10.000
coreside	27,529	0.494	0.500	0.000	1.000

### 2.5 Research methodology

#### 2.5.1 Fixed effects regression

This paper uses a fixed effects regression to measure the relationship between PA and cognitive function. This article fixed the year and province effects, clustered on the village-level administrative units, while incorporating demographic and sociological characteristics of the sample to refine the model.

#### 2.5.2 Propensity score matching

This paper employs robustness checks through the substitution of explanatory variables and propensity score matching (PSM) to ensure that the empirical regression results are more reliable (26). Using nearest neighbor matching and radius matching with a radius of 0.05, PSM divided the samples into a processing group (participating in physical activity) and a control group (not participating in physical activity) to make it similar to a natural experiment, thereby reducing selection bias and better observing the net effect of physical activity participation on cognitive function in middle-aged and older adult people.

#### 2.5.3 Heterogeneity analysis

This paper also employs heterogeneity analysis to explore the impact of physical activity on the cognitive function of middle-aged and older adult populations. We grouped the middle-aged and older adult population based on household registration (rural or not), gender, coreside of children, and marital status, conducting separate regressions to test whether the impact of physical activity on their cognitive function varies.

## 3 Results

### 3.1 Benchmark regression results

Table 2 presents the estimation results of the benchmark regression. Column (1) does not include control variables and fixed effects to avoid potential “bad controls (27).” Column (2) includes individual demographic and sociological characteristics as control variables in the regression. Column (3) adds time and regional fixed effects, and the results remain significant. This paper selects the results from Column (3) as the benchmark. The estimation results indicate that participation in physical activity significantly enhances the cognitive function of middle-aged and older adult individuals, suggesting that physical activity can improve cognitive abilities in this age group, consistent with previous research. From the control variables, cognitive function declines with age, males generally have better cognition function than females, and middle-aged and older adult individuals in urban areas have stronger cognitive function compared to those in rural areas. Healthier individuals tend to have better cognitive function (28, 29). Individuals with higher education levels also have better cognitive function (30), and there may be a life-cycle interaction between the two: individuals with stronger cognitive function have better learning abilities, leading to better educational attainment, which in turn further enhances their cognitive abilities (31). An interesting finding is that marriage can improve the cognitive function of middle-aged and older adult individuals within a family, but the presence of children may lead to cognitive decline in the older adult. This can be linked to informal care: existing literature suggests that more informal care may reduce the health of the older adult, so policies promoting informal care should be implemented with caution (32). For couples, their companionship is a process of mutual support and care, where both are caregivers and care recipients, thus improving the cognitive function of both partners; however, when there are more children, especially when they are present, the closest or cohabiting children are often the main caregivers in the family, as shown by existing literature (33). Therefore, after older adult individuals receive sufficient care from their children, their self-care needs may decrease, potentially leading to a decline in self-care abilities, which is reflected in cognitive decline.

**Table 2:** Benchmark regression results (*N* = 27,529).

	(1)	(2)	(3)
	Cognition	Cognition	Cognition
PA	1.157^***^	0.608^***^	0.617^***^
	(0.138)	(0.120)	(0.087)
age		−0.114^***^	−0.121^***^
		(0.005)	(0.004)
gender		0.018	0.088
		(0.098)	(0.097)
health		0.222^***^	0.193^***^
		(0.028)	(0.024)
marriage		0.458^***^	0.440^***^
		(0.075)	(0.077)
education		1.364^***^	1.234^***^
		(0.104)	(0.073)
rural		−1.189^***^	−1.144^***^
		(0.077)	(0.078)
smoking		−0.215^***^	−0.274^***^
		(0.058)	(0.063)
drinking		0.091	0.099
		(0.094)	(0.074)
hchild		−0.135^***^	−0.066^*^
		(0.042)	(0.037)
coreside		−0.209^***^	−0.022
		(0.056)	(0.048)
_cons	6.634^***^	12.688^***^	12.992^***^
	(0.145)	(0.300)	(0.281)
*N*	27,529	27,529	27,529
*R* ^2^	0.007	0.178	0.215
adj. *R*^2^	0.007	0.178	0.214
province	NO	NO	YES
year	NO	NO	YES

Standard errors in parentheses.

^*^p < 0.1, ^**^p < 0.05, ^***^p < 0.01, the same applies hereinafter.

### 3.2 Robustness test

Table 3 column (1) lists the substitution dependent variable as the empirical result of personal self-assessment of memory ability. As mentioned earlier, memory ability is a major component of cognitive function, therefore self-assessed memory can serve as a good alternative explanatory variable in this context. This paper categorizes samples with self-assessed memory scores of 1as poor, otherwise as good. Empirical results show that physical activity significantly improves the memory ability of the older adult, and the regression results remain significant at the 10% level. This result strongly supports the basic conclusion of this paper: physical activity can improve the cognitive function of the older adult. Since self-assessment reports generally underestimate the effectiveness (34), this result may be underestimated, that is, in fact, the effect may be better.

**Table 3:** Robustness test results.

	(1)	(2)	(3)	(4)
	Memory	Serial7s	Cognition	Cognition
PA	0.023^**^	0.215^***^	1.065^***^	0.618^***^
	(0.010)	(0.037)	(0.175)	(0.073)
age	−0.002^***^	−0.018^***^	−0.119^***^	−0.120^***^
	(0.001)	(0.002)	(0.009)	(0.003)
gender	0.113^***^	0.565^***^	0.259	0.093
	(0.011)	(0.041)	(0.197)	(0.062)
health	0.099^***^	0.038^***^	0.065	0.189^***^
	(0.003)	(0.007)	(0.063)	(0.021)
marriage	−0.024^**^	0.183^***^	0.414^**^	0.439^***^
	(0.009)	(0.037)	(0.196)	(0.068)
education	0.047^***^	0.346^***^	1.009^***^	1.231^***^
	(0.007)	(0.022)	(0.195)	(0.051)
rural	−0.097^***^	−0.359^***^	−1.038^***^	−1.144^***^
	(0.008)	(0.028)	(0.173)	(0.053)
smoking	−0.029^***^	−0.061^*^	−0.308^*^	−0.274^***^
	(0.007)	(0.031)	(0.187)	(0.059)
drinking	0.005	0.018	0.153	0.094^**^
	(0.009)	(0.019)	(0.155)	(0.048)
hchild	−0.010^***^	−0.038^***^	−0.097^*^	−0.066^***^
	(0.003)	(0.012)	(0.055)	(0.019)
coreside	0.015^***^	0.001	0.217	−0.022
	(0.005)	(0.022)	(0.135)	(0.042)
_cons	0.534^***^	3.901^***^	12.794^***^	11.823^***^
	(0.040)	(0.112)	(0.758)	(0.244)
*N*	27,529	27,529	2,791	27,438
*R* ^2^	0.105	0.107	0.206	0.214
adj. *R*^2^	0.104	0.106	0.194	0.213
province	YES	YES	YES	YES
year	YES	YES	YES	YES

Column (2) use represents performance in the serial 7’s to value their cognition. Serial 7’s is a quick and reasonably accurate test for assessing intellectual efficiency or deterioration in patients with psychiatric and neurologic disorders (35). Interviewers ask the respondent to subtract 7 from 100 and then continue subtracting 7 from each answer for 4 more trials. Correct subtractions are based on the previous answer given, the more times respondents make the right calculation, the higher they score. The empirical results show that PA significantly improved the accuracy of serial7’s calculation for middle-aged and older adults at the 1% level, further corroborating the improvement in cognitive function due to physical activity.

Columns (3) and (4) of Table 3 are the PSM estimation results using nearest neighbor matching and radius matching with a radius of 0.05, respectively. In order to alleviate the problem of selection bias, on the basis of changing the form of variable setting, this paper further uses PSM to construct a counterfactual causal framework to test the causal effect between physical activity and cognitive function. PSM divided the samples into a processing group (participating in physical activity) and a control group (not participating in physical activity) to make it similar to a natural experiment, thereby reducing selection bias and better observing the net effect of physical activity participation on cognitive function in middle-aged and older adult people. To this end, this paper uses nearest neighbor matching and caliper matching to match the processing group and the control group, respectively. Then a balance test is carried out to test the validity of the match based on the “common support hypothesis” and the “independence hypothesis” to ensure that there are no systematic differences in explanatory variables other than cultural predisposition between the control group and the treatment group, and to ensure that the treatment process is similar to a natural experiment to obtain more realistic results. After matching, the standardized deviation of most variables is less than 10%. The t-test shows that there is no significant systematic difference between most of the treatment group and the control group, so it meets the basic requirements of randomized experiments. It can be found from the table that after using the PSM method to reduce the selection bias, the net effect is still positive, which shows that the results of the basic regression are robust.

Under various settings, the basic conclusions of this paper have remained stable. For the middle-aged and older adult, the cognitive function of the groups that participate in physical activity is stronger than that of the groups that do not participate in physical activity.

### 3.3 Heterogeneity analysis

Table 4 presents the results of the heterogeneity regression in this paper. The first and second columns of Table 4 show the urban–rural regression results, while the third and fourth columns display the gender regression results, the fifth and sixth columns show the results of the cohabitation with children regression, and the seventh and eighth columns present the results of the heterogeneity analysis by marital status. Empirical results indicate that physical activity significantly improves cognitive function among middle-aged and older adult residents regardless of whether they reside in urban or rural areas, regardless of gender, and regardless of whether they are accompanied by children. This suggests that the implementation of policies to promote physical activity may achieve cognitive function improvement for all older adult individuals. Given the increasing aging population in China today, active aging and healthy aging are imperative. Therefore, the government can enhance the conditions for older adult individuals to participate in physical activity by configuring public facilities, thereby improving the cognitive function of the older adult population (36).

**Table 4:** Heterogeneity analysis.

	Rural		Gender		Coreside		Marriage	
	(1)	(2)	(3)	(4)	(5)	(6)	(7)	(8)
PA	0.546^***^	0.858^***^	0.537^***^	0.690^***^	0.468^***^	0.751^***^	0.598^***^	0.723^***^
	(0.079)	(0.163)	(0.095)	(0.120)	(0.121)	(0.107)	(0.090)	(0.179)
age	−0.129^***^	−0.099^***^	−0.122^***^	−0.119^***^	−0.116^***^	−0.127^***^	−0.121^***^	−0.120^***^
	(0.005)	(0.008)	(0.006)	(0.005)	(0.005)	(0.006)	(0.005)	(0.007)
gender	0.229^*^	−0.293^**^			0.101	0.084	0.077	0.239
	(0.116)	(0.141)			(0.087)	(0.135)	(0.102)	(0.219)
health	0.170^***^	0.261^***^	0.205^***^	0.174^***^	0.175^***^	0.207^***^	0.195^***^	0.143^**^
	(0.024)	(0.042)	(0.029)	(0.030)	(0.029)	(0.038)	(0.025)	(0.058)
marriage	0.445^***^	0.515^**^	0.440^***^	0.457^***^	0.372^***^	0.536^***^		
	(0.072)	(0.196)	(0.104)	(0.104)	(0.114)	(0.090)		
education	1.445^***^	1.068^***^	0.964^***^	1.731^***^	1.182^***^	1.267^***^	1.209^***^	1.486^***^
	(0.119)	(0.067)	(0.090)	(0.118)	(0.112)	(0.069)	(0.074)	(0.282)
smoking	−0.263^**^	−0.350^***^	−0.241^***^	−0.442^***^	−0.357^***^	−0.202^**^	−0.264^***^	−0.362
	(0.101)	(0.104)	(0.086)	(0.145)	(0.102)	(0.078)	(0.067)	(0.220)
drinking	0.081	0.142	0.126	0.062	0.055	0.150	0.093	0.184
	(0.082)	(0.119)	(0.091)	(0.081)	(0.091)	(0.097)	(0.077)	(0.114)
hchild	0.016	−0.278^***^	−0.020	−0.095^**^	−0.049	−0.079^*^	−0.079^*^	−0.009
	(0.042)	(0.044)	(0.044)	(0.035)	(0.060)	(0.045)	(0.041)	(0.050)
coreside	0.038	−0.184^*^	−0.120^*^	0.079			−0.040	0.134
	(0.063)	(0.092)	(0.067)	(0.062)			(0.050)	(0.108)
rural			−1.067^***^	−1.183^***^	−1.026^***^	−1.281^***^	−1.124^***^	−1.327^***^
			(0.085)	(0.111)	(0.101)	(0.113)	(0.072)	(0.224)
_cons	11.887^***^	12.264^***^	13.382^***^	12.365^***^	12.848^***^	13.273^***^	13.507^***^	12.534^***^
	(0.297)	(0.566)	(0.393)	(0.339)	(0.337)	(0.418)	(0.312)	(0.684)
*N*	20,519	7,010	13,996	13,533	13,612	13,917	24,587	2,942
*R* ^2^	0.177	0.239	0.202	0.237	0.208	0.227	0.203	0.230
adj. *R*^2^	0.175	0.235	0.199	0.235	0.206	0.225	0.202	0.219
province	YES	YES	YES	YES	YES	YES	YES	YES
year	YES	YES	YES	YES	YES	YES	YES	YES

### 3.4 Mechanism analysis

This study has used fixed effects regression and propensity score matching (PSM) to strengthen causal inference. To add depth to the findings, this study measures the impact of physical activity on cardiovascular health in the older adult population based on answers to the questionnaire regarding whether they have ever had hypertension. Empirical results in Table 5 show that engaging in physical activity significantly reduces the risk of hypertension in middle-aged and older adult populations. Among the potential targets for enhancing cognitive health in older adults, arterial hypertension stands out as one of the most common and modifiable pathologies (37). Hypertension has been shown to exert adverse effects on several cognitive domains, including abstract reasoning and/or executive function, memory, and mental processing speed (38). Thus, participation in physical activity can improve cardiovascular health in middle-aged and older adults, thereby slowing the progression of cognitive decline.

**Table 5:** Mechanism analysis.

	Hypertension
PA	−0.025^*^
	(0.014)
age	0.009^***^
	(0.001)
gender	−0.003
	(0.013)
health	−0.081^***^
	(0.005)
marriage	−0.001
	(0.012)
education	−0.001
	(0.011)
rural	−0.048^***^
	(0.010)
smoking	−0.019
	(0.011)
drinking	0.026^***^
	(0.008)
hchild	−0.005
	(0.004)
coreside	−0.002
	(0.007)
_cons	0.114^*^
	(0.058)
*N*	27,529
*R* ^2^	0.091
adj. *R*^2^	0.089
province	YES
year	YES

## 4 Discussion

In the previous sections, we use fixed effects regression of time and region to confirm the role of PA in improving cognitive function in middle-aged and older adult individuals. After replacing the explained variable with self-rated memory and serial7s results, the conclusion remains valid, while the PSM estimation results further reinforced the reliability of this perspective. Heterogeneity analysis shows that this conclusion is applicable to middle-aged and older adult populations, indicating that policies encouraging physical activity among the older adult can comprehensively improve their cognitive function, thereby enhancing the practical significance of this study. Finally, we attempt to explain the mechanism behind this conclusion, with regression results showing that physical activity significantly reduces the hypertension risk in middle-aged and older adult individuals, thereby improving their cognitive function.

### 4.1 The effects of different intensities of physical activity on cognitive function

Table 6 displays the regression results of different levels of physical activity intensity on residents’ cognitive function. This paper categorizes residents’ physical activity intensity into three levels based on CHARLS responses: Low, Moderate, and High. Low-intensity physical activities (LPA) represents mild activities such as walking for leisure. Moderate-intensity physical activities (MPA) represents activities that make people breathe faster than usual (carrying light stuff, bicycling at a normal speed, Tai-Chi, and speed walking, etc.). Vigorous-intensity physical activities (VPA) represents activities that cause shortness of breath (carrying heavy stuff, digging, bicycling at a fast speed, etc.)

**Table 6:** Different intensities of physical activity.

	(1)	(2)	(3)
	Cognition	Cognition	Cognition
LPA	0.416^***^		
	(0.055)		
MPA		0.422^***^	
		(0.038)	
VPA			−0.138^**^
			(0.051)
age	−0.122^***^	−0.119^***^	−0.124^***^
	(0.004)	(0.004)	(0.005)
gender	0.091	0.130	0.064
	(0.096)	(0.095)	(0.096)
health	0.198^***^	0.193^***^	0.228^***^
	(0.024)	(0.024)	(0.030)
marriage	0.441^***^	0.427^***^	0.458^***^
	(0.076)	(0.078)	(0.074)
education	1.231^***^	1.225^***^	1.341^***^
	(0.073)	(0.073)	(0.102)
rural	−1.131^***^	−1.164^***^	−1.171^***^
	(0.078)	(0.077)	(0.078)
smoking	−0.274^***^	−0.273^***^	−0.224^***^
	(0.063)	(0.063)	(0.059)
drinking	0.104	0.092	0.053
	(0.072)	(0.073)	(0.085)
hchild	−0.066^*^	−0.067^*^	−0.089^**^
	(0.037)	(0.037)	(0.040)
coreside	−0.022	−0.020	−0.081
	(0.048)	(0.048)	(0.059)
_cons	13.264^***^	13.263^***^	13.665^***^
	(0.262)	(0.261)	(0.343)
*N*	27,529	27,529	27,529
*R* ^2^	0.215	0.216	0.198
adj. *R*^2^	0.214	0.215	0.198
province	YES	YES	YES
year	YES	YES	YES

The regression results show that moderate and low-intensity physical activity significantly improve residents’ cognitive function, while high-intensity physical activity significantly reduces residents’ cognitive function at the 5% level. This empirical finding can be interpreted as “too much of a good thing.” Existing literature indicates that occupational athletes have weaker cognitive abilities in their later years compared to other age groups (39). Vigorous exercise acutely elevates reactive oxygen species (ROS) production beyond antioxidant defenses. Lu et al. found that high-intensity cycling increased plasma 8-OHdG (a DNA oxidative damage marker) by 37%, which correlated with reduced hippocampal functional connectivity (40).

For residents, high-intensity physical activity may harm brain health (41), ultimately resulting in a reduction in residents’ cognitive function. Therefore, it is evident that moderate physical activity is beneficial for middle-aged and older adult populations, while high-intensity physical activity may not be worth the cost. Consequently, it is advisable to provide more recreational and leisure-oriented low to moderate-intensity exercise facilities for this group.

### 4.2 The effects of physical activity on different types of cognitive functions

Table 7 shows the effects of physical activity on cognitive function types. Column (1) lists the effects of physical activity on residents’ immediate memory ability, while Column (2) shows the effects on residents’ delayed memory ability. Empirical results indicate that PA significantly improves residents’ cognitive function at the 1% level for all types of cognitive functions. This finding further reinforces the basic conclusion of this paper. An interesting discovery is that the number of children may reduce older adult people’s memory function. This might reflect the dependency pathway of the middle-aged and older adult on their children’s care: older adult people may rely on their children’s reminders for memory. When older adult people get used to this memory assistance, their own functions may deteriorate. Consequently, this paper provides new empirical evidence for the argument in previous studies that informal care may affect the health of the older adult.

**Table 7:** Different types of cognitive functions.

	(1)	(2)
	Imrecall	Dlrecall
PA	0.290^***^	0.327^***^
	(0.046)	(0.048)
age	−0.048^***^	−0.072^***^
	(0.002)	(0.002)
gender	0.027	0.061
	(0.039)	(0.063)
health	0.074^***^	0.119^***^
	(0.011)	(0.015)
marriage	0.234^***^	0.206^***^
	(0.043)	(0.041)
education	0.570^***^	0.664^***^
	(0.036)	(0.041)
rural	−0.522^***^	−0.622^***^
	(0.034)	(0.049)
smoking	−0.119^***^	−0.156^***^
	(0.026)	(0.044)
drinking	0.036	0.063
	(0.036)	(0.043)
hchild	−0.039^**^	−0.026
	(0.017)	(0.021)
coreside	−0.018	−0.005
	(0.021)	(0.031)
_cons	5.937^***^	7.055^***^
	(0.145)	(0.162)
*N*	27,529	27,529
*R* ^2^	0.195	0.256
adj. *R*^2^	0.194	0.255
province	YES	YES
year	YES	YES

### 4.3 The effects of physical activity on cognitive function in older adults

In today’s increasingly aging society, directly identifying the effects of physical activity on cognitive function in the older adult has strong practical significance. Table 8 regression results show that participation in physical activity can effectively improve cognitive function in the older adult. To ensure the reliability of the empirical results and avoid ambiguity due to the definition of the older adult population, Column (1) is a regression for individuals aged 60 and above, while Column (2) is a regression for individuals aged 65 and above. Currently, LTC policies worldwide face issues of fiscal and caregiver shortages (42). Improving the health of the older adult can delay their institutionalization, reduce long-term care needs, and improve their health conditions, thereby alleviating government fiscal pressure and promoting the optimization of LTC resource allocation (43). Therefore, advocating for moderate physical activity participation among the older adult can effectively promote healthy aging, particularly enhancing their self-care abilities. The improvement in the older adult’s self-care abilities can alleviate family caregiving burdens and social care burdens, thus better addressing aging issues (44). The empirical results in Table 7 largely align with the benchmark regression, also indicating the robustness of the basic conclusions presented in this paper.

**Table 8:** Older adults.

	(1)	(2)
	Cognition	Cognition
PA	0.708^***^	0.684^***^
	(0.106)	(0.133)
age	−0.105^***^	−0.134^***^
	(0.007)	(0.009)
gender	0.142	0.194
	(0.145)	(0.147)
health	0.151^***^	0.159^***^
	(0.038)	(0.049)
marriage	0.480^***^	0.410^***^
	(0.086)	(0.090)
education	1.242^***^	1.258^***^
	(0.100)	(0.115)
rural	−1.320^***^	−1.349^***^
	(0.122)	(0.150)
smoking	−0.202^*^	−0.224^*^
	(0.109)	(0.110)
drinking	0.082	0.045
	(0.107)	(0.106)
hchild	−0.075^*^	−0.040
	(0.040)	(0.044)
coreside	0.051	0.118
	(0.052)	(0.075)
_cons	11.966^***^	14.003^***^
	(0.555)	(0.752)
*N*	13,465	8,312
*R* ^2^	0.150	0.155
adj. *R*^2^	0.147	0.151
province	YES	YES
year	YES	YES

## 5 Limitation

However, there are several limitations that must be acknowledged. First, the presence of unknown confounding factors could have influenced the results, despite the statistical adjustments made in the observational study. Second, the self-reporting of physical activity intensity per week and memory function introduces subjectivity, which may lead to biased estimates. Third, the biennial CHARLS assessments (2011–2018) may overlook critical short-term cognitive transitions. Neuroplastic research suggests exercise-induced cognitive changes typically develop over 4–8 months, whereas CHARLS’ two-year intervals cannot capture. This temporal mismatch may underestimate both the risks of abrupt activity changes and early cognitive decline signals. Future studies could enhance temporal resolution by integrating longitudinal surveys with monthly mobile cognitive tests and wearable activity monitoring for high-risk subgroups.

## 6 Conclusion and implications

The present study provides robust evidence that physical activity (PA) significantly enhances cognitive function among the older adult population in China. This finding aligns with a growing body of international research that underscores the positive impact of PA on cognitive health in later life. For instance, Firth et al. (45) demonstrated that regular aerobic exercise increases the size of the hippocampus, a brain region critical for learning and memory, thereby improving cognitive performance in older adults. Similarly, a meta-analysis by Warsh et al. (46) concluded that exercise interventions have a significant positive effect on cognitive function in adults over 50 years of age. The results of our study suggest that PA not only improves global cognitive function but also specifically enhances executive functions and memory, which are particularly vulnerable to age-related decline. This is consistent with the findings of Colcombe et al. (15), who reported that older adults who engaged in regular physical activity showed significant improvements in executive control processes. Moreover, the study by Bherer et al. (47) highlighted that both aerobic and resistance training can lead to cognitive benefits in older adults, further supporting the notion that different types of PA can be effective in maintaining cognitive health.

The implications of these findings are far-reaching. Given the increasing burden of dementia and cognitive impairment on healthcare systems and societies worldwide, promoting PA among the older adult could be a cost-effective and accessible intervention to mitigate these challenges. As highlighted by the World Health Organization (48), regular physical activity is a key component of healthy aging and can reduce the risk of chronic diseases, including cognitive decline. Therefore, public health policies should prioritize initiatives that encourage older adults to engage in regular PA, such as community-based exercise programs, accessible recreational facilities, and educational campaigns about the benefits of PA for cognitive health. Furthermore, the study’s findings emphasize the importance of tailoring PA interventions to individual needs and preferences. While moderate-intensity PA appears to be beneficial for most individuals, the potential negative effects of high-intensity PA on cognitive function suggest that a personalized approach is warranted. This aligns with the recommendations of the American College of Sports Medicine (49), which advocates for exercise prescriptions that consider individual fitness levels, health status, and personal goals.

In conclusion, the study underscores the critical role of PA in maintaining and enhancing cognitive function among the older adult. By integrating PA into the daily lives of older adults, societies can potentially reduce the incidence of cognitive impairment, delay the onset of dementia, and improve the overall quality of life for this demographic. Future research should continue to explore the optimal types, intensities, and durations of PA for cognitive health, as well as the mechanisms underlying these effects. Additionally, further investigation into the role of social and environmental factors in facilitating PA participation among the older adult is warranted to inform the development of comprehensive and effective public health strategies.

## Data Availability

The original contributions presented in the study are included in the article/supplementary material, further inquiries can be directed to the corresponding author.

## References

[1] Klimova B Dostalova R. The impact of physical activities on cognitive performance among healthy older individuals. Brain Sci. (2020) 10:377. doi: 10.3390/brainsci10060377, PMID: 32560126 PMC7349640

[2] Peng D. Negative population growth and population ageing in China. China Popul Dev Stud. (2023) 7:95–103. doi: 10.1007/s42379-023-00138-z, PMID: 40103751

[3] Erickson KI Donofry SD Sewell KR Brown BM Stillman CM. Cognitive aging and the promise of physical activity. Annu Rev Clin Psychol. (2022) 18:417–42. doi: 10.1146/annurev-clinpsy-072720-014213, PMID: 35044793

[4] Mariani E Monastero R Mecocci P. Mild cognitive impairment: a systematic review. J Alzheimers Dis. (2007) 12:23–35. doi: 10.3233/JAD-2007-12104, PMID: 17851192

[5] Mitchell AJ Shiri-Feshki M. Rate of progression of mild cognitive impairment to dementia – meta-analysis of 41 robust inception cohort studies. Acta Psychiatr Scand. (2009) 119:252–65. doi: 10.1111/j.1600-0447.2008.01326.x, PMID: 19236314

[6] Sun M Mainland BJ Ornstein TJ Mallya S Fiocco AJ Sin GL. The association between cognitive fluctuations and activities of daily living and quality of life among institutionalized patients with dementia. Int J Geriatr Psychiatry. (2018) 33:e280–5. doi: 10.1002/gps.4788 28940504

[7] Duan J. “Association of cognitive impairment and elderly mortality: differences between two cohorts ascertained 6-years apart in China,” BMC Geriatr. (2020) 20:29. doi: 10.1186/s12877-020-1424-4 31992221 PMC6988297

[8] Atchison T Massman P Doody R. Baseline cognitive function predicts rate of decline in basic-care abilities of individuals with dementia of the Alzheimer’s type. Arch Clin Neuropsychol. (2007) 22:99–107. doi: 10.1016/j.acn.2006.11.006, PMID: 17174522

[9] Song R Fan X Seo J. Physical and cognitive function to explain the quality of life among older adults with cognitive impairment: exploring cognitive function as a mediator. BMC Psychol. (2023) 11:51. doi: 10.1186/s40359-023-01087-5, PMID: 36814329 PMC9948328

[10] Hu S Ge J Fang M Yang J. Role of intergenerational connections in cognitive aging: evidence from a Chinese longitudinal study. Front Public Health. (2024) 12:1396620. doi: 10.3389/fpubh.2024.1396620, PMID: 39234093 PMC11371578

[11] Iso-Markku P Aaltonen S Kujala UM Halme HL Phipps D Knittle K. Physical activity and cognitive decline among older adults: a systematic review and meta-analysis. JAMA Netw Open. (2024) 7:e2354285. doi: 10.1001/jamanetworkopen.2023.54285, PMID: 38300618 PMC10835510

[12] Ingold M Tulliani N Chan CCH Liu KPY. Cognitive function of older adults engaging in physical activity. BMC Geriatr. (2020) 20:229. doi: 10.1186/s12877-020-01620-w, PMID: 32616014 PMC7333382

[13] Yang Z Hotterbeex P Marent P-J Cerin E Thomis M Van Uffelen J. Physical activity, sedentary behaviour, and cognitive function among older adults: a bibliometric analysis from 2004 to 2024. Ageing Res Rev. (2024) 97:102283. doi: 10.1016/j.arr.2024.102283, PMID: 38552882

[14] Angevaren M Aufdemkampe G Verhaar H Aleman A Vanhees L. Physical activity and enhanced fitness to improve cognitive function in older people without known cognitive impairment. Cochrane Database Syst Rev. (2008) 3:CD005381. doi: 10.1002/14651858.CD005381.pub3 18646126

[15] Colcombe SJ Erickson KI Raz N Webb AG Cohen NJ McAuley E. Aerobic fitness reduces brain tissue loss in aging humans. J Gerontol A Biol Sci Med Sci. (2003) 58:M176–80. doi: 10.1093/gerona/58.2.M176, PMID: 12586857

[16] Festa F Medori S Macrì M. Move your body, boost your brain: the positive impact of physical activity on cognition across all age groups. Biomedicines. (2023) 11:1765. doi: 10.3390/biomedicines11061765 37371860 PMC10296541

[17] Erickson KI Voss MW Prakash RS Basak C Szabo A Chaddock L. Exercise training increases size of hippocampus and improves memory. Proc Natl Acad Sci. (2011) 108:3017–22. doi: 10.1073/pnas.1015950108, PMID: 21282661 PMC3041121

[18] Erickson KI Hillman C Stillman CM Ballard RM Bloodgood B Conroy DE. Physical activity, cognition, and brain outcomes: a review of the 2018 physical activity guidelines. Med Sci Sports Exerc. (2019) 51:1242–51. doi: 10.1249/MSS.0000000000001936, PMID: 31095081 PMC6527141

[19] Hötting K Röder B. Beneficial effects of physical exercise on neuroplasticity and cognition. Neurosci Biobehav Rev. (2013) 37:2243–57. doi: 10.1016/j.neubiorev.2013.04.005, PMID: 23623982

[20] Barha CK Davis JC Falck RS Nagamatsu LS Liu-Ambrose T. Sex differences in exercise efficacy to improve cognition: a systematic review and meta-analysis of randomized controlled trials in older humans. Front Neuroendocrinol. (2017) 46:71–85. doi: 10.1016/j.yfrne.2017.04.002, PMID: 28442274

[21] Covenant A Yates T Rowlands AV Dempsey PC Edwardson CL Hall AP. Replacing sedentary time with sleep and physical activity: associations with physical function and wellbeing in type 2 diabetes. Diabetes Res Clin Pract. (2024) 217:111886. doi: 10.1016/j.diabres.2024.111886, PMID: 39369857

[22] Huang Y Loux T Huang X Feng X. The relationship between chronic diseases and mental health: a cross-sectional study. Ment Health Prev. (2023) 32:200307. doi: 10.1016/j.mhp.2023.200307, PMID: 40104671

[23] Duan S Chen D Wang J Paramboor MS Xia Z Xu W. Digital exclusion and cognitive function in elderly populations in developing countries: insights derived from 2 longitudinal cohort studies. J Med Internet Res. (2024) 26:e56636. doi: 10.2196/56636, PMID: 39546790 PMC11607572

[24] Wang X Hu P Ai Y Zhou S Li Y Zhou P. Dual group-based trajectories of physical activity and cognitive function in aged over 55: a nationally representative cohort study. Front Public Health. (2024) 12:1450167. doi: 10.3389/fpubh.2024.1450167, PMID: 39534743 PMC11554534

[25] Mátyás L Nerlove ML. The econometrics of multi-dimensional panels: theory and applications In: Mátyás L. (editor) Advanced studies in theoretical and applied econometrics, no. 54. 2nd ed. Cham: Springer International Publishing (2024). doi: 10.1007/978-3-031-49849-7

[26] McMurry TL Hu Y Blackstone EH Kozower BD. Propensity scores: methods, considerations, and applications in the journal of thoracic and cardiovascular surgery. J Thorac Cardiovasc Surg. (2015) 150:14–9. doi: 10.1016/j.jtcvs.2015.03.057, PMID: 25963441

[27] Cinelli C Forney A Pearl J. A crash course in good and bad controls. Sociol Methods Res. (2024) 53:1071–104. doi: 10.1177/00491241221099552

[28] Levinthal BR Morrow DG Tu W Wu J Murray MD. Cognition and health literacy in patients with hypertension. J Gen Intern Med. (2008) 23:1172–6. doi: 10.1007/s11606-008-0612-2, PMID: 18459011 PMC2517973

[29] Federman AD Sano M Wolf MS Siu AL Halm EA. Health literacy and cognitive performance in older adults. J Am Geriatr Soc. (2009) 57:1475–80. doi: 10.1111/j.1532-5415.2009.02347.x, PMID: 19515101 PMC2754116

[30] Barnes LL Mendes De Leon CF Wilson RS Bienias JL Evans DA. Social resources and cognitive decline in a population of older African Americans and whites. Neurology. (2004) 63:2322–6. doi: 10.1212/01.WNL.0000147473.04043.B3, PMID: 15623694

[31] Lövdén M Fratiglioni L Glymour MM Lindenberger U Tucker-Drob EM. Education and cognitive functioning across the life span. Psychol Sci Public Interest. (2020) 21:6–41. doi: 10.1177/1529100620920576, PMID: 32772803 PMC7425377

[32] Wang Y Yang W Avendano M. Does informal care reduce health care utilisation in older age? Evidence from China. Soc Sci Med. (2022) 306:115123. doi: 10.1016/j.socscimed.2022.115123, PMID: 35724586

[33] Bergeot J. Care for elderly parents: do children cooperate? J Popul Econ. (2024) 37:6. doi: 10.1007/s00148-024-00992-2, PMID: 40103751

[34] Sitzmann T Johnson SK. When is ignorance bliss? The effects of inaccurate self-assessments of knowledge on learning and attrition. Organ Behav Hum Decis Process. (2012) 117:192–207. doi: 10.1016/j.obhdp.2011.11.004

[35] Karzmark P. Validity of the serial seven procedure. Int J Geriatr Psychiatry. (2000) 15:677–9. doi: 10.1002/1099-1166(200008)15:8<677::AID-GPS177>3.0.CO;2-4, PMID: 10960879

[36] Duan Y Wagner P Zhang R Wulff H Brehm W. Physical activity areas in urban parks and their use by the elderly from two cities in China and Germany. Landsc Urban Plan. (2018) 178:261–9. doi: 10.1016/j.landurbplan.2018.06.009

[37] Ungvari Z Toth P Tarantini S Prodan CI Sorond F Merkely B. Hypertension-induced cognitive impairment: from pathophysiology to public health. Nat Rev Nephrol. (2021) 17:639–54. doi: 10.1038/s41581-021-00430-6, PMID: 34127835 PMC8202227

[38] Iadecola C Yaffe K Biller J Bratzke LC Faraci FM Gorelick PB. Impact of hypertension on cognitive function: a scientific statement from the American Heart Association. Hypertension. (2016) 68:e67–94. doi: 10.1161/HYP.0000000000000053, PMID: 27977393 PMC5361411

[39] Symons IK Bruce L Main LC. Impact of overtraining on cognitive function in endurance athletes: a systematic review. Sports Med Open. (2023) 9:69. doi: 10.1186/s40798-023-00614-3, PMID: 37552398 PMC10409951

[40] Lu Y Wiltshire HD Baker JS Wang Q. Effects of high intensity exercise on oxidative stress and antioxidant status in untrained humans: a systematic review. Biology. (2021) 10:1272. doi: 10.3390/biology10121272, PMID: 34943187 PMC8698973

[41] Li Y Fu C Song H Zhang Z Liu T. Prolonged moderate to vigorous physical activity may lead to a decline in cognitive performance: a Mendelian randomization study. Front Aging Neurosci. (2024) 16:1403464. doi: 10.3389/fnagi.2024.1403464, PMID: 39372647 PMC11449848

[42] Ariaans M Linden P Wendt C. Worlds of long-term care: a typology of OECD countries. Health Policy. (2021) 125:609–17. doi: 10.1016/j.healthpol.2021.02.009, PMID: 33715875

[43] Yang C-H Chen Y-C Hsu W Chen Y-H. Evaluation of smart long-term care information strategy portfolio decision model: the national healthcare environment in Taiwan. Ann Oper Res. (2023) 326:505–36. doi: 10.1007/s10479-023-05358-7, PMID: 37361069 PMC10148017

[44] Lawless MT Tieu M Feo R Kitson AL. Theories of self-care and self-management of long-term conditions by community-dwelling older adults: a systematic review and meta-ethnography. Soc Sci Med. (2021) 287:114393. doi: 10.1016/j.socscimed.2021.114393, PMID: 34534780

[45] Firth J Stubbs B Vancampfort D Schuch F Lagopoulos J Rosenbaum S. Effect of aerobic exercise on hippocampal volume in humans: a systematic review and meta-analysis. Neuroimage. (2018) 166:230–8. doi: 10.1016/j.neuroimage.2017.11.007, PMID: 29113943

[46] Walsh EI Smith L Northey J Rattray B Cherbuin N. Towards an understanding of the physical activity-BDNF-cognition triumvirate: a review of associations and dosage. Ageing Res Rev. (2020) 60:101044. doi: 10.1016/j.arr.2020.101044, PMID: 32171785

[47] Langlois F Vu TTM Chasse K Dupuis G Kergoat M-J Bherer L. Benefits of physical exercise training on cognition and quality of life in frail older adults. J Gerontol B Psychol Sci Soc Sci. (2013) 68:400–4. doi: 10.1093/geronb/gbs069, PMID: 22929394

[48] World Health Organization. Global action plan on physical activity 2018–2030: more active people for a healthier world. Geneva: World Health Organization, (2018). [Online]. Available: https://iris.who.int/handle/10665/272722 (Accessed March 27, 2025).

[49] American College of Sports Medicine. ACSM’s guidelines for exercise testing and prescription, 9. ed. Baltimore, Md.: Wolters Kluwer, Lippincott Williams & Wilkins, (2014).

